# The Dose Response of Taurine on Aerobic and Strength Exercises: A Systematic Review

**DOI:** 10.3389/fphys.2021.700352

**Published:** 2021-08-18

**Authors:** Qi Chen, Zheng Li, Ricardo A. Pinho, Ramesh C. Gupta, Ukadike C. Ugbolue, Anand Thirupathi, Yaodong Gu

**Affiliations:** ^1^Faculty of Sports Science, Ningbo University, Ningbo, China; ^2^Laboratory of Exercise Biochemistry in Health, Graduate Program in Health Sciences, School of Medicine, Pontifícia Universidade Católica do Paraná, Curitiba, Brazil; ^3^School of Agricultural Sciences and Rural Development (SASRD), Nagaland University, Medziphema, India; ^4^School of Health and Life Sciences, University of the West of Scotland, Blantyre, United Kingdom

**Keywords:** taurine, exercise, muscle, oxidative stress, antioxidants

## Abstract

Taurine is a naturally occurring amino acid involved in various functions, including regulating ion channels, cell volume, and membrane stabilization. However, how this molecule orchestrates such functions is unknown, particularly the dose response in exercised muscles. Therefore, this review aimed to systematically review the dose response of taurine on both aerobic and strength exercise performance. In accordance with the Preferred Reporting Items for Systematic Reviews and Meta-Analyses (PRISMA) statement, relevant articles were sought on PubMed, Medline, Web of Science, and Google Scholar using related terms, including taurine, exercise performance, exercise, muscle, physical training, running, strength, endurance exercise, resistance exercise, aerobic exercise, and swimming. Ten articles were retrieved, reviewed, and subjected to systematic analysis. The following parameters were used to assess exercise performance in the selected studies: creatine kinase (CK), lactic acid dehydrogenase, carbohydrate, fat, glycerol, malondialdehyde, enzymatic antioxidants, blood pH, taurine level, and muscular strength. From the selected literature, we observed that taurine supplementation (2 g three times daily) with exercise can decrease DNA damage. Furthermore, 1 g of acute taurine administration before or after exercise can decrease lactate levels. However, acute administration of taurine (6 g) at a high dose before the start of exercise had no effect on reducing lactate level, but increased glycerol levels, suggesting that taurine could be an effective agent for prolonged activities, particularly at higher intensities. However, further studies are warranted to establish the role of taurine in fat metabolism during exercise. Finally, we observed that a low dose of taurine (0.05 g) before performing strength enhancing exercises can decrease muscular fatigue and increase enzymatic antioxidants.

**Systematic Review Registration:**http://www.crd.york.ac.uk/PROSPERO, PROSPERO (CRD42021225243).

## Introduction

Taurine is a prominent free amino acid with little or no role in protein synthesis. However, it has multiple physiological roles, including interacting with ion channels, membrane stabilization, and cellular osmoregulation (Shihabi et al., 1989; Schaffer and Kim, 2018). The intracellular concentration ranges of taurine range approximately between 5 and 20 μmol/g in many tissues, particularly in excitable tissues, including skeletal muscle, heart, and brain (De Luca et al., 2015). The main sources of dietary taurine are meat, shellfish, sea vegetables, and dairy products. The biosynthesis of taurine is as follows: First, cysteine is oxidized by cysteine dioxygenase to form cysteine sulfinic acid. Next, decarboxylation of cysteine sulfinic acid by cysteine sulfinic acid decarboxylase occurs, producing hypotaurine; finally, the oxidation of hypotaurine to taurine. This pattern may be affected by the person's nutritional intake and the availability of its precursor cysteine (Ripps and Shen, 2012; De Luca et al., 2015). Exercise is a crucial factor that can influence the pattern of biosynthesis of taurine by oxidizing the precursor cysteine. For example, high-intensity-induced redox modification of cysteine can undergo several post-translational modifications to produce either muscular adaptation or fatigue, which affects taurine biosynthesis (Thirupathi et al., 2020). This scenario can demand additional taurine requirements from external sources. Taurine a potential role in preventing oxidative damage and restoring muscle function in muscular disorders (Ripps and Shen, 2012; De Luca et al., 2015; Thirupathi et al., 2020). Furthermore, taurine-regulated calcium homeostasis can increase calcium-binding proteins during muscle contraction, resulting in greater muscle strength and endurance (El Idrissi and Trenkner, 1999; Galloway et al., 2008; Thirupathi et al., 2018; Wen et al., 2019).

A person's physical condition and fitness level may also influence the availability of taurine in skeletal muscle (Henriksson, 1991; Graham et al., 1995). This may be due to structural differences in an individual's physical exercise threshold. There are marked differences between trained and untrained individuals, that is, after prolonged exercise, induced amino acid changes show higher levels of taurine in trained individuals (Henriksson, 1991; Graham et al., 1995). Animal studies have shown that taurine can regulate excitation contraction by altering chloride conductance (De Luca et al., 1996), and taurine knockout mice exhibit myofibril derangements consistent with exercise capacity impairment (Ito et al., 2010). Nor can we omit the role of taurine in energy metabolism, as shown in other studies (Mozaffari et al., 1986; Wen et al., 2019). For example, rats fed with the taurine inhibitor beta-alanine can stimulate the rate of glycolysis and lactate production, but this effect was independent of taurine depletion (Mozaffari et al., 1986; Gladden, 2004). However, this study did not verify the role of taurine during exercise. Exercise with higher intensities can affect the redox status of cells, but taurine may catalyze the positive effect of exercise by replenishing the thiol pool in skeletal muscle, as previously reported (Silva et al., 2011). Exercise induced thiol-disulfide exchange activates various redox-sensitive signaling pathways, including PGC-1 alpha, which can greatly influence taurine biosynthesis. Although taurine improved the contractile properties of muscle by reducing muscle soreness and oxidative damage (Hamilton et al., 2006; De Carvalho et al., 2017), the concentration of taurine required to elicit a clinically relevant improvement in muscle function remains ambiguous. Since taurine is described in the literature as one of the safest amino acids to use, the exact dosage and duration of effectiveness in humans requires careful attention. Furthermore, gaps in understanding the role of supplements such as taurine in exercise performance, remain significant. Therefore, we hypothesize that establishing a proper taurine dose will not only increase exercise performance, but also improve muscle function by altering various biochemical functions.

## Methods

### Search Strategy

In accordance with the guidelines for the Preferred Reporting Items for Systematic Reviews and Meta-Analyses (PRISMA) statement, a search for relevant articles was carried out on PubMed, Medline, ISI, Web of Science, and Google Scholar using a broad range of synonyms and related terms, namely taurine, exercise performance, exercise, muscle, physical training, running, strength, endurance exercise, resistance exercise, aerobic exercise, and swimming. To avoid the risk of missing relevant articles, additional papers were sought in the gray literature (i.e., generic Web search) and through the bibliography of previous reviews. One author ran the search and screened the initial titles after duplicates were removed. Other authors independently examined the search results in depth for potentially relevant articles. We included only papers published in peer-reviewed journals that reported findings from experimentally controlled human studies. We excluded articles such as unpublished papers, conference posters, or those reporting findings of non-experimental studies (e.g., pre-/post-intervention studies, case series, etc.). The first author's name, year of publication, sample of intervention, design and duration of the study, topic, type of intervention, outcome, assessment, and results were recorded using an electronic spreadsheet.

### Inclusion and Exclusion Criteria

We performed the PICO acronym (participants, interventions, comparisons, outcomes) for inclusion, and the criteria are shown in Table 1. We included taurine studies involving exercise performance if they met the following eligibility criteria: (1) studies reporting participants on exercise programs, including running, swimming, and cycling with taurine supplementation; (2) search outputs that included only articles that were peer-reviewed and published in English language journals; (3) only parameters that were related to exercise performance were included as types of outcome measures, including oxygen uptake, oxidative stress, DNA damage, and energy metabolism parameters, including fat oxidation and glycerol levels.

**Table 1:** Eligibility criteria in accordance with PICO (Participants, interventions, comparators, outcomes).

**Domain**	**Inclusion criteria**
Participants (P)	Healthy athletes and volunteers
Intervention (I)	Taurine
Comparators (C)	Endurance or strength exercise or placebo
Outcomes (O)	Taurine supplementation decreases DNA damage, oxidative stress and muscular fatigue and increase the fat oxidation to improve exercise performance

### Full-Text Articles Selection

The abstracts of the articles were further refined using the following criteria: we included prospective cohort studies, cross-sectional studies, and randomized clinical trials. We excluded animal studies that used taurine supplementation to improve exercise performance.

### Risk of Bias Assessment

The risk of bias assessment was independently performed by two authors based on the Cochrane risk of bias assessment tool. A third author was consulted if any disagreement arose.

### Data Extraction and Analysis

For each study, the study characteristics (e.g., authors, nationality of the first author and published year), participant characteristics (e.g., number of participants, age, and gender), and intervention (taurine supplementation) were extracted and identified by an author and verified by another author based on taurine improving exercise performance. All parameters were evaluated based on blood and urine samples collected during or after the exercise program. Disagreements were resolved through discussion with other authors.

## Results

### Search Results

**Figure 1:**
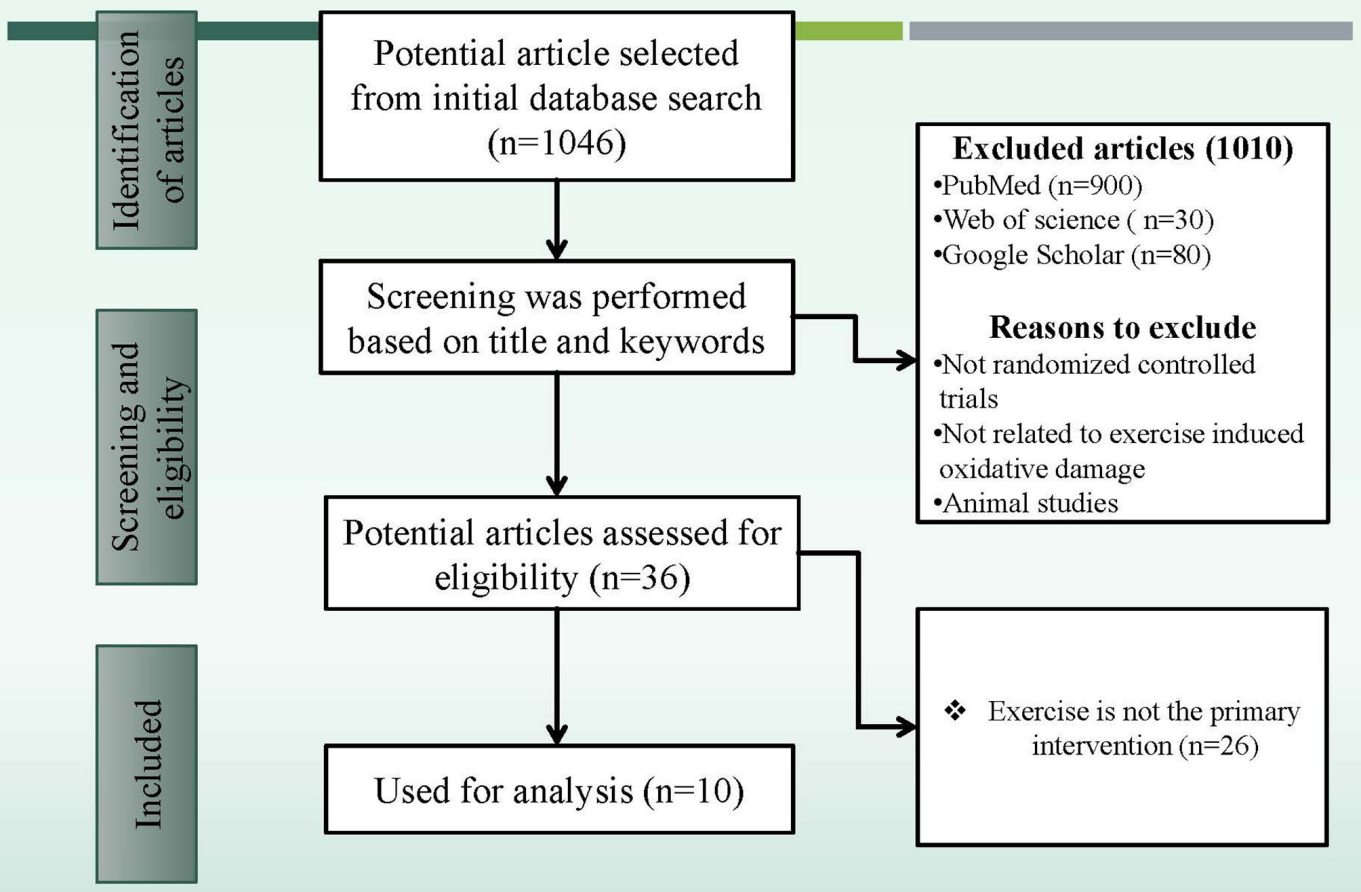
The search flowchart for screening process.

After a general search through PubMed, Scopus, and Google Scholar, 1,046 articles were identified from the initial database searches presented in Figure 1. After initial screening, the database was created based on the results of the selected articles' results (Table 2) and a total of 1,010 articles were excluded. The remaining 36 potential article abstracts were carefully evaluated, and a further 26 articles were excluded. The full text of the remaining 10 articles was retrieved, reviewed, and then included for systematic analysis.

**Table 2:** Number of publications in exclusion criteria based on its main topic.

**Publication main topic**	**Number of publications**
Publications including non-human studies	900
Publications that are not in English with other purpose, unpublished papers and conference papers	13
Publications that are not related to taurine and exercise performance	120

### Study Outcome

All the selected studies used taurine as supplements.

### Risk of Bias in Included Studies

**Figure 2:**
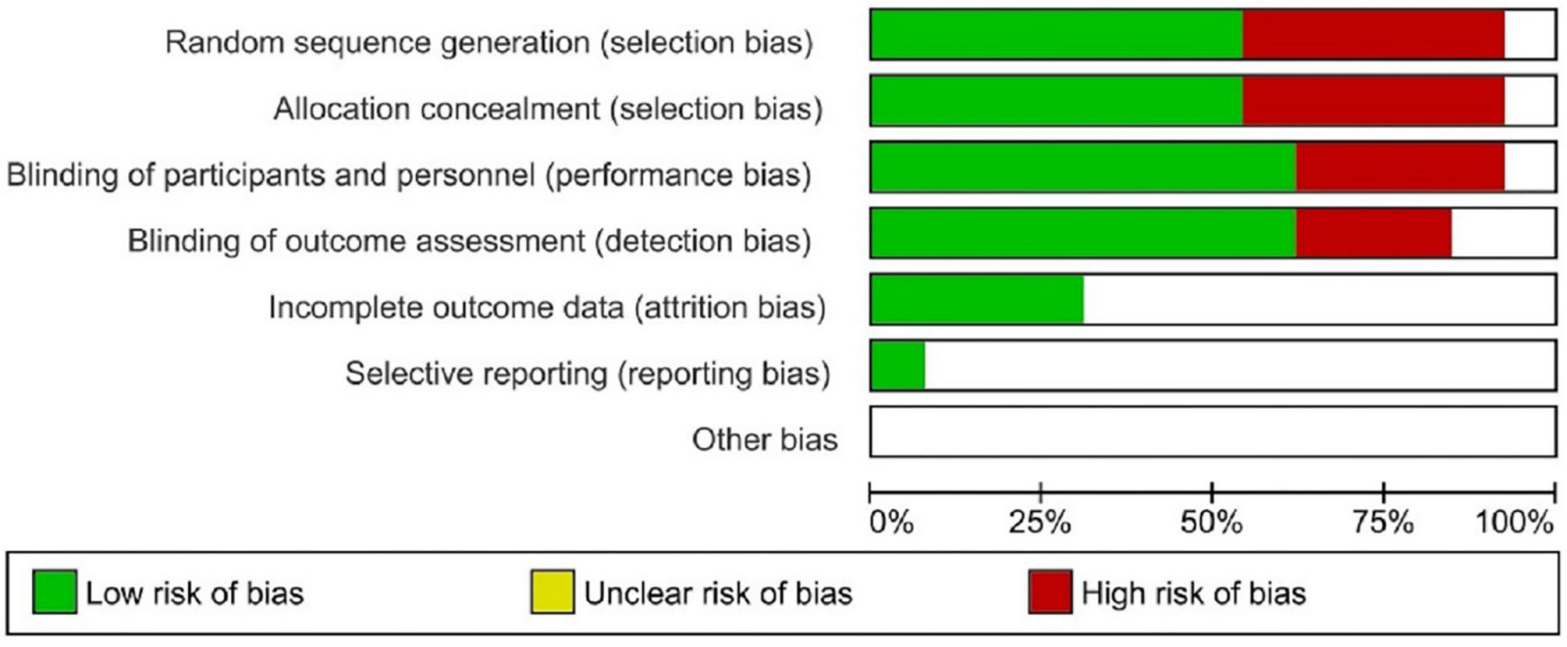
Risk of bias evaluation of included studies.

Of the 10studies included for systematic review, at least five had a risk of bias. Four studies had a high risk of blinding of the participants and outcome assessments (Table 3). Five studies had a high risk of randomization, and one had allocation concealment. All included studies had an unclear or low risk of incomplete outcome, selective reporting, and other forms of bias (Figure 2).

**Table 3:** Risk of bias evaluation of included studies.

**References**	**Random sequence generation**	**Allocation concealment**	**Blinding of participants and personnel**	**Blinding of outcome assessment**	**Incomplete outcome data**	**Selective reporting**	**Other bias**
De Carvalho et al. (2017)	Low	Low	Low	Low	Unclear	Unclear	Unclear
Carvalho et al. (2020)	Low	Low	Low	Low	Unclear	Unclear	Unclear
Galan et al. (2018)	low	Low	Low	Low	Unclear	Unclear	Unclear
da Silva et al. (2014)	Low	Low	Low	Low	Unclear	Unclear	Unclear
Kowsari et al. (2018)	Unclear	Unclear	Low	Low	Unclear	Unclear	Unclear
Balshaw et al. (2013)	Low	Low	Low	Low	Low	Unclear	Unclear
Ward et al. (2016)	High	High	High	High	Unclear	Unclear	Unclear
Milioni et al. (2016)	Low	Low	Low	Low	Unclear	Unclear	Unclear
Rutherford et al. (2010)	Low	Low	Low	Low	Unclear	Unclear	Unclear
Zhang et al. (2004)	High	High	High	Unclear	Unclear	Unclear	Unclear

### Exercise Performance Analysis With Taurine Administration

Although various roles of taurine have been reported in muscle, there is no specific mechanism reported, to-date, about taurine that can improve muscle performance and decrease muscle damage. Therefore, this review systematically analyzed the taurine dose response of improved exercise performance and decreasing muscle damage. For this reason, we selected 10studies that used taurine as a supplement to improve exercise performance (Table 4).

**Table 4:** The study characteristics of included studies.

**References**	**Study population**	**Study design**	**Sex**	**No. of participants**	**Exercise type**		**Dose and time of taurine administration**	**Assessment**	**Main results**
					**Endurance exercise**	**Strength exercise**			
De Carvalho et al. (2018)	Brazil	Randomized double-blind crossover study (Athletes received taurine or Placebo)	(Male athletes; aged 19.78 ± 3.38)	9	Participants performed two 400-m front crawl maximal efforts with an interval of 1 week between swimming efforts	–	Acute taurine supplementation (6g) received before 120 min of swimming	Taurine↑ Lactate ↔ Glycerol↑	Taurine did not improve the athlete performance, but it increased the glycerol level the in plasma.
Carvalho et al. (2020)	Brazil	Randomized double-blind crossover study (Subjects received taurine or Placebo)	(Male subjects; aged 24.8 ± 4.07)	17	Aerobic exercise was performed on a treadmill at 60% of VO_2max_	–	Two different doses of taurine supplementation (6g or 3 g) received before the exercise	6 g of Taurine↑ 3 g of Glycerol↑ 6 g of Glycerol ↔ 6 g of Lipid oxidation ↑ No difference in lipid oxidation between 3 g and 6 g	6 g of taurine increased the lipid oxidation and 3 g of taurine increased glycerol level, but no differences between 6 and 3 g of taurine in increasing lipid oxidation
Galan et al. (2018)	Brazil	Randomized double-blind crossover study (Subjects received taurine or Placebo)	(Male long-distance triathletes; aged 30 ± 4.6)	9	Participants performed treadmill running with a constant incline of 1% and an initial speed of 8 km.h^−1^ and it was increased at every three minutes until the exhaustion	–	3g/day of taurine received immediately after exercise for 8 weeks	CK↑ LDH↔ TNF-α ↔ IL-6↔ Taurine↑	Increase in CK level and no significant difference in IL-6, Taurine and TNF-α
da Silva et al. (2014)	Brazil	Randomized double-blind crossover study (Subjects received taurine supplementation or Placebo)	(Male volunteers aged 21 ± 6)	21	–	Participants performed elbow flexion and extension on the Scott bench at an intensity of 80% of 1RM	0.05g of taurine supplementation before the exercise	CK↓ LDH↓ Isometric strength ↑ Muscular fatigue ↓ SOD↑ CAT↑ GPx↑ TNF-α ↔	Taurine supplementation decreased muscle damage, oxidative stress, but not inflammation.
Kowsari et al. (2018)	Iran	Randomized study (Subjects received taurine supplementation or Placebo)	(Male squash players aged 23 ± 1.41)	20	All the Subjects had practiced at a competition level, for 30 minutes/ 4 days a week for 6 months.	–	1g/5times/ a day of taurine supplement taken before and after the submaximal activity	Lactate↓	The study results observed taurine is effective in reducing neuromuscular fatigue, choice reaction time, and blood lactate accumulation
Balshaw et al. (2013)	UK	Randomized double-blind crossover study (Subjects received taurine or Placebo)	(Male runners aged 19.9 ± 1.2)	8	The participants completed 3 kilometer running on a treadmill. Participants completed a 10 mins standardized pace warm-up (2-min at 10 km h^−1^, 3min at 12 km h^−1^ and 5min 14 km h^−1^)	–	1g/day taurine received before the exercise	Lactate↔ Heart rate↔ Ratings of perceived excretion↔	Taurine significantly enhanced the endurance running performance, but the mechanism is unclear.
Ward et al. (2016)	UK	Randomized double-blind crossover study (Subjects received taurine or Placebo)	(Male trained cyclists aged 34.6 ± 11.5)	11	All participants were male cyclists and performed 4-km time trials	–	1g of taurine received before 2 hours of performing exercise	Lactate ↑ pO2↑, pCO2↓, pH↓ and HCO3↓^−^ VO_2_↔ Heart rate↔	Taurine did not give any performance advantage during 4km.
Milioni et al. (2016)	Brazil	Randomized crossover study (Subjects received taurine supplementation or Placebo)	(Male trainees; aged 25 ± 6)	17	Participants performed treadmill running until voluntary exhaustion at submaximal intensity (7.5 km.h-1).	–	6g of taurine received 90 min prior to the exhaustive test	Lactate↔ VO_2_↔ Maximal accumulated oxygen deficit↔	Taurine did not improve the high intensity running program.
Rutherford et al. (2010)	Canada	Randomized crossover study (Subjects received taurine supplementation or Placebo)	(Male athletes; aged 27.2 ± 1.5)	11	The participants underwent a continuous incremental cycle test to exhaustion to determine the maximal pulmonary oxygen uptake	-	1.66g of taurine received 1 hour before the exercise	VO_2_↔ VCO_2_↔ CHO↔ Fat↑	Taurine did not enhance the time trial performance, but significantly increased the fat oxidation.
Zhang et al. (2004)	Japan	–	(Male college students aged 18– 20)	11	All subjects performed exercise task on a bicycle ergometer with an increased workload of 20W/min. The subjects had 4min warming up exercise of 0W/min to reach the required speed.	–	After the first exercise test, 2g of taurine three times per day for 7 days prior to the second exercise test	TBARS ↔ Exercise time↑ Heart rate↑ VO_2_↑	Taurine may attenuate exercise-induced DNA damage and improve the exercise capacity which may be due to its cellular protective properties.

*CK, creatine kinase; LDH, lactic acid dehydrogenase; TNF-α, tumor necrosis factor-α; IL-6, interlukin-6; HCO3, bicarbonate; CO2, carbon dioxide; CHO, carbohydrate; TBARS, thiobarbituric acid reactive substances; MDA, malondialdehyde; CAT, catalase; GPx, glutathione peroxidase*.

### Aerobic Exercise Performance With Taurine

Among the 10 studies, nine used taurine supplementation to improve aerobic exercise performance. One study assessed the effects of taurine combined with low-fat chocolate milk supplementation, after exercise, on markers of muscle damage and inflammatory responses, including interleukin-6 (IL-6) and tumor necrosis factor-α (TNF-α) (Galan et al., 2018). Aerobic exercise-induced inflammatory markers have both positive and negative effects, including increased muscle damage. For example, aerobic exercise can release muscle-derived IL-6 into the circulation to maintain glucose homeostasis (Howlett et al., 1999; Pedersen et al., 2001). In contrast, elevated levels of TNF-α induce the formation of reactive oxygen species (ROS) and reduce muscle function, depending on exercise type and intensity (Mangner et al., 2013). In this study, taurine did not elevate IL-6 levels during endurance training, which implies that taurine has little or no role in IL-6-induced benefits (Mangner et al., 2013). However, endurance training showed that TNF-α levels did not significantly increase with taurine supplementation (Galan et al., 2018). This may be due to exercise type, intensity, duration, or the quantity of taurine administered. The blood lactate response to exercise is considered to be a normal variable to measure exercise performance. From the selected literature, six studies assessed blood lactate levels with taurine supplementation in endurance training (Balshaw et al., 2013; Milioni et al., 2016; Ward et al., 2016; De Carvalho et al., 2018; Kowsari et al., 2018). These studies demonstrated that acute taurine supplementation of 6 g/week before performing exercise had no effect on reducing blood lactate levels in endurance training, such as running or swimming (Milioni et al., 2016; De Carvalho et al., 2018), but it increased glycerol levels (De Carvalho et al., 2018). One g of acute taurine administration 5 times daily before and after exercise significantly decreased blood lactate levels (Kowsari et al., 2018). Different doses of taurine have different effects on increasing fat oxidation. For example, one study reported that 1.66 g of acute taurine administered before exercise increased fat oxidation (Rutherford et al., 2010), while a separate study reported that 6 g of taurine supplementation before a single bout of fasting aerobic exercise increased fat oxidation, but 3 g of taurine did not increase fat oxidation (Carvalho et al., 2020). After the first exercise, participants received 2 g of taurine three times daily during endurance training and exhibited decreased DNA damage (Zhang et al., 2004).

### Strength Exercise Performance With Taurine

Strength exercise is more often characterized by a loading profile that combines high force and low fiber recruitment, which induces substantial mechanical stress, resulting in disruption of sarcomere contraction and excitation system failure. This scenario initiates injuries, including various biochemical and inflammatory alterations, and an increase in reactive oxygen species. One study used taurine supplementation (0.05 g) per day for 14 days and then performed eccentric strength exercise for 21 days with taurine supplementation. Taurine has been reported to improve enzymatic antioxidants and decrease muscular fatigue by reducing oxidative stress (da Silva et al., 2014). However, da Silva et al. (2014) reported that taurine did not have any effect on inflammation.

## Discussion

This review systematically reported the dose response of taurine in improving exercise performance. We found from the selected literature that endurance training requires a higher dose of taurine, ~1 g five times daily, to prevent muscle-related damage whereas strength exercise requires a lower dose of taurine (0.05 g) to increase enzymatic antioxidants and decrease muscular fatigue.

### Effects of Taurine Supplementation on Endurance Exercise Performance

Endurance performance is dependent not only on taurine dose, but also on age, gender, and duration of exercise (Balshaw et al., 2013; Ward et al., 2016; De Carvalho et al., 2018; Galan et al., 2018). Creatine kinase and blood lactate accumulation are important indicators for assessing muscle damage (Baird et al., 2012; Manojlović and Erčulj, 2019). From the selected literature, we observed that acute taurine supplementation (~1 g 5 times daily before and after exercise) can effectively reduce blood lactate levels during endurance training (Kowsari et al., 2018), while taurine supplementation before exercise (1 g) increased lactate levels during endurance training (Ward et al., 2016). However, acute administration of higher doses of taurine (6 g) during high-intensity endurance training had no effect on blood lactate levels (Milioni et al., 2016). This may be due to the blood buffering capacity of taurine (Nakada et al., 1992; Balshaw et al., 2013; Kowsari et al., 2018). Endurance training with taurine supplementation of 3 g daily immediately after exercise increased CK (Galan et al., 2018), which may be due to initial muscle damage caused by endurance exercise (Brancaccio et al., 2006). From these studies, we suggest that different exercise types require different doses of taurine to effectively improve exercise performance. Furthermore, 6 g of taurine per week can improve lipid metabolism by increasing glycerol in the plasma and muscle glycogen storage, indicating that high doses of taurine could be useful during high-intensity endurance exercises (De Carvalho et al., 2018). The intake of taurine at ~3,000–10,000 mg/day is safe for humans (Shao and Hathcock, 2008). The highest dose of taurine in humans in the pre-exercise condition was 3,000 mg (Eudy et al., 2013). Some studies have reported that taurine dose does not affect exercise performance (Zhang et al., 2004; Milioni et al., 2016; Ward et al., 2016; De Carvalho et al., 2018), suggesting that further studies regarding the taurine dose response of exercise, are warranted.

### Effect of Taurine on Strength Exercise Performance

Strength exercise requires conflicting actions by which muscle length is increased while increasing mechanical stress. As a result, microstructural damage and further reduction in muscle contraction and excitation system occurs, and increased ROS production and activation of inflammatory cascades are also evident. Studies, including those undertaken by our group, have shown that taurine can increase muscle function, decrease oxidative damage, and increase antioxidants during strength exercise (Ra et al., 2016; Thirupathi et al., 2018). However, the mechanism by which taurine protects muscles from oxidative stress, remains unknown. Taurine decreases delayed oxidative stress and attenuates arterial stiffness after eccentric exercise through its antioxidant properties (Ra et al., 2016). This may be due to the role of taurine in nitric oxide production (Ra et al., 2016). Furthermore, taurine mitigates inflammation-induced secondary damage, which facilitates muscle recovery during strength exercise. We observed from the reviewed literature that taurine improves muscular strength and reduces muscle fatigue by reducing oxidative stress. However, there was no change in the inflammation status, which could be related to the intensity and duration of exercise. Other studies have also observed that taurine prevents oxidative stress without activating inflammation cascades during exercise (Khazaei, 2012; Shirvani et al., 2012).

### Role of Taurine on Metabolism

**Figure 3:**
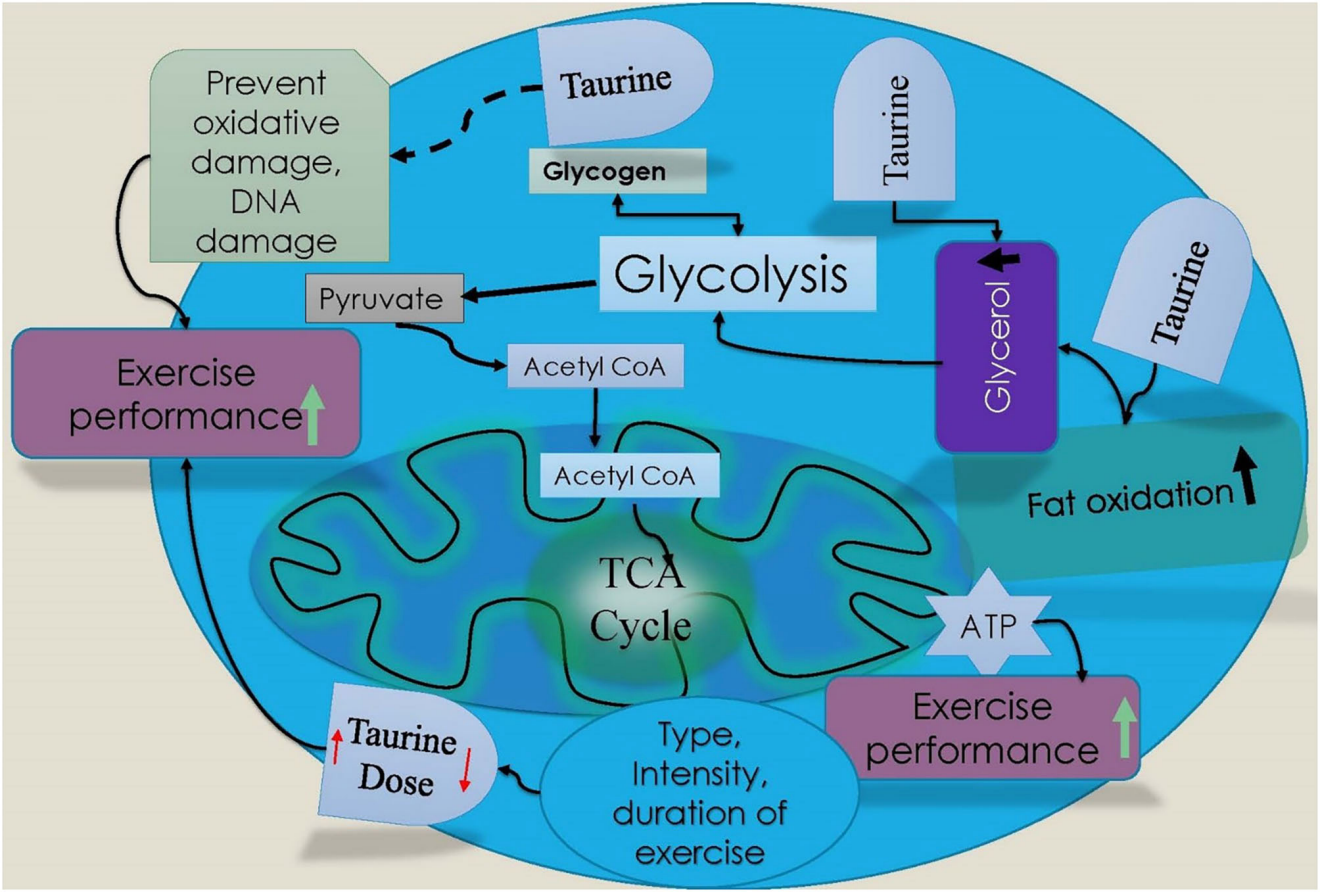
Schematic diagram of taurine effect on exercise performance. Taurine is involved increase in fatty acid oxidation and glycerol during exercise, which could increase performance during high intensity exercise. Taurine‘s (Red arrow marks indicate taurine dose increase or decrease for exercise performance) role in increasing exercise performance depends on type, intensity, and duration of exercise.

Taurine improves metabolism, and taurine deficiency may reduce metabolic activity (Wen et al., 2019). Therefore, taurine could be a useful candidate for improving exercise performance because it may increase fat oxidation by altering intracellular and extracellular metabolic environments (Wolfe, 1998; de Bari et al., 2002; Zhang et al., 2004; Galloway et al., 2008) (Figure 3). Rutherford and colleagues have shown that taurine supplementation (1.66 g ingestion) increased fat oxidation by up to 16% in athletes during exercise, compared to placebo, and this may be due to the activation of adenylate cyclase and cyclic adenosine monophosphate (AMPc) (Shepherd et al., 1981; Rutherford et al., 2010; Pistor et al., 2014). Exercise with taurine improves energy expenditure and white adipose tissue metabolism, which supports the regulation of both body weight and exercise performance time. For example, De Carvalho et al., reported that exercise with taurine supplementation increased resting energy expenditure and released blood irisin in obese women (De Carvalho et al., 2021). De Carvalho et al. (2018) reported that taurine increases glycerol levels in plasma and improves lipolysis in healthy individuals (De Carvalho et al., 2018). Taurine supplementation with exercise may also increase mitochondrial activity and fatty acid oxidation by altering the gene expression of various enzymes and proteins, including peroxisome proliferator-activated receptor gamma coactivator 1-alpha (PGC-1α), lipoprotein lipase, acyl-CoA synthase, and acyl-CoA oxidase (ACOX), which are linked to lipid substrate oxidation (Murakami, 2015; Jia et al., 2016). De Carvalho et al. (2018) showed that taurine supplementation with exercise improved lipid metabolism by increasing mitochondrial respiratory capacity, uncoupling protein 1 (UCP1), and uncoupling protein 2 (UCP2), and increased the expression of fat oxidation-related genes, including aconitase 2 (ACO2) and ACOX1 in obese women (De Carvalho et al., 2021). These studies suggest a potential role for taurine in the regulation of energy metabolism in both active and obese individuals.

### Limitation

A few factors that could limit the complete benefit of taurine in improving exercise performance include type of exercise, taurine dose, ingestion strategy, time of administration, biosynthesis, and antioxidant properties (Schaffer and Kim, 2018). For example, different doses elicit different effects during exercise. A high dose of taurine before exercise only had an effect on increasing glycerol, whereas taurine administration before strength exercises or during and after endurance exercise effectively reduced muscle damage by reducing DNA damage and oxidative stress. However, these studies did not evaluate direct DNA damage and oxidative damage markers, and this could be a limitation of those studies. Furthermore, a lower dose of taurine administered before undertaking strength exercise decreases muscle damage by attenuating oxidative stress, and this may be due to strength exercise-induced ROS formation, thereby preconditioning the exercise to adaptation. However, this must be established with studies involving a larger number of participants. In addition, excess availability of taurine as an antioxidant may interfere with the activity of mitochondrial respiration and adaptation induced by exercise. This scenario could limit the exercise-induced benefits, and this must be studied by fixing the taurine dose as low as possible, otherwise, it could limit the redox signaling benefits. Focusing on these aspects may effectively warrant the use of taurine to improve exercise performance.

### Future Directions

For the past two decades, researchers have shown that exercise-induced benefits are linked to a person's nutritional uptake and health status. Taurine is a sulfur-containing free amino acid present in almost all excitable tissues, with concentration ranging from 5 to 20 μm/g. Since taurine was discovered in 1827, there has been much research investigating the mechanistic role of taurine in skeletal muscle and other systems, including antioxidation, Ca^2+^ homeostasis, and osmoregulation (Cozzoli et al., 2011; Terrill et al., 2016; Seidel et al., 2019). However, excess availability of taurine may blunt the ROS-induced benefits, as mentioned earlier. Conversely, increased contractile properties of muscles, particularly during high-intensity exercises, may affect the release of taurine into muscle fibers, especially when taurine depletion occurs among fast-twitch fibers, which ultimately compromises exercise performance (Galler et al., 1990; Tallon et al., 2007). In this scenario, taurine supplementation could be useful to compensate for the large loss of taurine. However, taurine administration as a supplement before, after, or during exercise, has a different effect, particularly on reducing muscle damage or muscle fatigue, which is a hallmark of limiting muscle performance and this correct dosage must be established by further study. However, designing and prescribing doses of taurine for effective and convincing outcomes remains difficult. Amino acids require several other supplemental conditions because of their first-pass effects and solubility constraints in crossing cell membranes. However, taurine is now regarded as an essential amino acid and perhaps a pharmaconutrient. In the future, further investigation may result in supportive evidence leading to taurine as a novel agent for such activities. Furthermore, taurine supplementation affects its concentration in blood plasma, and increased levels of taurine may be an indicator of muscle damage, muscle fatigue, or osmolarity adaptation in the whole blood, which can be misinterpreted during or after exercise. Therefore, establishing a method to determine specific taurine threshold values in the plasma that can either indicate muscle damage or are sensitive enough to increase its uptake to retain its cellular level, is a worthwhile study for the future. It is also important to explore methods that can increase muscle taurine concentrations following taurine supplementation. This may be achieved through the manipulation of dietary content or by establishing a method that could increase the cellular uptake of taurine by the specific transporter molecule taurine transporter (TauT) during exercise. One study has reported that exercise suppresses the expression of TauT (Han et al., 2006), future work focusing on increasing the availability of TauT in order to retain an optimal taurine concentration within cells is warranted.

## Conclusions

This review systematically reviewed the effects of taurine on exercise performance. From the selected literature, different doses of taurine can increase lipid metabolism and decrease DNA damage during aerobic exercise. For example, acute administration of taurine (1.66 g) before performing exercise can increase lipid metabolism, and even higher doses of taurine (6 g) supplementation before a single bout of exercise can increase lipid metabolism. However, 3 g of taurine had no impact on lipid metabolism, which may be due to differences in the taurine kinetics of various tissues, which may fluctuate the release of taurine into the blood and further increase in blood taurine. Furthermore, after the first exercise, 2 g of taurine, 3 times a day, can decrease DNA damage. Regarding strength exercise, a low dose of taurine supplementation (0.05 g) with strength exercise improves enzymatic antioxidants and muscular fatigue by reducing exercise-induced oxidative stress.

## Data Availability Statement

The original contributions presented in the study are included in the article/Supplementary Material, further inquiries can be directed to the corresponding authors.

## Author Contributions

AT, QC, ZL, and YG conceived the original idea, developed the framework, and wrote the manuscript. AT, RP, UU, RG, and YG provided critical feedback and contributed to the final version. All authors were involved in the final direction of the paper and contributed to the final version of the manuscript. All authors have read and agreed to the published version of the manuscript.

## Conflict of Interest

The authors declare that the research was conducted in the absence of any commercial or financial relationships that could be construed as a potential conflict of interest.

## Publisher's Note

All claims expressed in this article are solely those of the authors and do not necessarily represent those of their affiliated organizations, or those of the publisher, the editors and the reviewers. Any product that may be evaluated in this article, or claim that may be made by its manufacturer, is not guaranteed or endorsed by the publisher.
