# Supplementary material for: The Dose Response of Taurine on Aerobic and Strength Exercises: A Systematic Review
Source: Front Physiol. 2021 Aug 18;12:700352. doi: 10.3389/fphys.2021.700352 (PMC8419774; doi:10.3389/fphys.2021.700352)
Supplement: Supplementary file 1 [file Data_Sheet_1.ZIP › Search strategy.docx]

**How Dose response of taurine can affect aerobic and strength exercises?**

PUBMED SEARCH KEYWORDS

| #1 Taurine and physiology | (("Taurine/metabolism"[Mesh]) AND ("Taurine/physiology"[Mesh] OR "Taurine/therapeutic use"[Mesh])) AND ("Taurine/physiology"[Mesh]) |
| --- | --- |
| #2 Exercise | ("Muscle Fibers, Skeletal"[Mesh]) AND ("Exercise"[Mesh]) |
| #3 Oxidative Stress | (("Oxidative Stress"[Mesh]) AND (("Muscle Fibers, Skeletal"[Mesh]) AND ("Exercise"[Mesh]))) AND ("Exercise"[Mesh]) |
| #4 oxidants | ("Taurine"[Mesh]) AND ("Oxidants"[Mesh]) |
| #5 Oxidative stress | ("Taurine"[Mesh]) AND ("Oxidative Stress"[Mesh]) |
| # 6 antioxidants | ("Taurine"[Mesh]) AND ("Antioxidants/physiology"[Mesh]) |
