# Supplementary material for: The Dose Response of Taurine on Aerobic and Strength Exercises: A Systematic Review
Source: Front Physiol. 2021 Aug 18;12:700352. doi: 10.3389/fphys.2021.700352 (PMC8419774; doi:10.3389/fphys.2021.700352)
Supplement: Supplementary file 1 [file Data_Sheet_1.ZIP › PRISMA_Checklist.docx]

| Topic | Check Item | Reported on Page |
| --- | --- | --- |
| Title | Identify the report as a systematic review, meta-analysis, or both. | 1 |
| ABSTRACT |  |  |
| Structured summary | Provided a structured summary including, as applicable: background; objectives; data sources; study eligibility criteria, participants, and interventions; study appraisal and synthesis methods; results; limitations; conclusions and implications of key findings | 1 |
| METHODS |  |  |
| Eligibility criteria | Abstract selection  Full-text articles selection  Risk of Bias Assessment  Data extraction and analysis | 2-3 |
| RESULTS |  |  |
|  | Search Results  Study Outcome  Risk of Bias of Included Studies  Synthesis of results | 3-11 |
| DISCUSSION |  |  |
|  | Summary of evidence | 11 |
|  | Limitations | 13 |
|  | Future direction | 14 |
|  | Conclusions | 14 |
